# Effects of irradiation on cumulative mortality in mice: shifting toward a younger age of death

**DOI:** 10.1093/jrr/rrad006

**Published:** 2023-02-10

**Authors:** Yuki Fujimichi, Michiya Sasaki, Kazuo Yoshida, Toshiyasu Iwasaki

**Affiliations:** Biology and Environmental Chemistry Division, Sustainable System Research Laboratory, Central Research Institute of Electric Power Industry, 1646 Abiko, Abiko-shi, Chiba 270-1194, Japan; Biology and Environmental Chemistry Division, Sustainable System Research Laboratory, Central Research Institute of Electric Power Industry, 1646 Abiko, Abiko-shi, Chiba 270-1194, Japan; Central Research Institute of Electric Power Industry, 2-11-1 Iwado kita, Komae-shi, Tokyo 201-8511, Japan; Strategy and Planning Division, Sustainable System Research Laboratory, Central Research Institute of Electric Power Industry, 2-11-1 Iwado kita, Komae-shi, Tokyo 201-8511, Japan

**Keywords:** shifting age of death, mice maintained for life, irradiation, all-cause mortality, tumor-related mortality, ovarian tumor

## Abstract

Recently, the question of whether cancer risk is only accelerated but not increased by radiation exposure has been raised. To explore this matter, we analyzed whether the cumulative mortality of irradiated mice could be explained by x-axis (age) shifted cumulative mortality of nonirradiated mice. We reanalyzed publicly available data on observed cumulative mortality or prevalence in irradiated female B6C3F_1_ mice that lived their entire lifespan. The results showed that the irradiated curve was well matched to uniformly shifted nonirradiated curve for the cumulative mortality of all causes of death but not for the cumulative mortality of all solid tumors and prevalence of ovarian tumors as is. After adjusting lifetime mortalities, it was also well matched for all solid and ovarian tumors. The shifted days by irradiation were 71–116 days for all causes of death, 56–135 days for all solid tumors, and 41–140 days for ovarian tumors in the 1.9 Gy-irradiated group. The response was switched between irradiation at 35 and 105 days consistently for all the above indexes, supporting the hypothesis that radiation sensitivity differs between juvenile and adults. The shifted days of all causes of death showed a tendency of linear response to dose. This concept of shifting the age of death can be applied not only for all cause of death but also for mortality of all solid tumors after adjusting the magnitude. These findings contribute to the discussion on the application of the ‘shifting age of death’ concept to radiation protection.

## INTRODUCTION

Excess relative risk (ERR) and excess absolute risk (EAR) are long-standing metrics for assessing radiation risk in epidemiological studies and radiation protection [1]. ERR is expressed as relative risk (RR) minus one, and RR is calculated as the ratio of the number of cases in the exposed population to that in the nonexposed population. EAR is calculated as the amount of excess cases against spontaneous cases. Recent research has highlighted the need of refocusing on the risk of developing irradiation-associated diseases at an early stage [2]. The hypothesis of ‘shifting the age of death’ has been considered for its usefulness in comparing risk due to radiation and other factors. Nakamura observed a 100-day shift in all-cause mortality for 8 Gy-irradiated B6C3F_1_ mice (21 mGy/day) and a 6-year shift in humans with 1 Gy radiation exposure at the age of 30 years [2]. There is, however, still insufficient discussion about the application of this ‘shifting the age of death’ concept to all solid tumors, which is important in radiation protection, and whether it can be applied over a wide range of doses.

Concerning these discussions about shifting the age of death, it is also important to expand the knowledge on mouse response, for which abundant data are available compared with those for humans. Epidemiological studies tracking the lives of people exposed to radiation are limited, but there are some data for mice maintained for life after irradiation under various conditions, such as dose, dose rate, and age at exposure. These experiments on maintaining mice for life have mainly been conducted in the Argonne National Laboratory [3–5], the National Institutes for Quantum and Radiological Science and Technology [6–12], and the Institute for Environmental Sciences [13]. Overall, the data set of B6C3F_1_ mice in Japan [6–13] is the most suitable to compare the effects between low and high dose rates.

To evaluate the applicability of the shifting the age of death concept, herein we reanalyzed published data on high dose-irradiated mice [6–8] concerning the effects of dose, and age at exposure on the shifting days for all cause of deaths, all solid tumors and site-specific tumors.

## MATERIALS AND METHODS

### Data of irradiated mice maintained throughout their lifespan

The cumulative mortalities of the open data set derived from female B6C3F_1_ mice irradiated at several doses (0.8–0.98 Gy/min) at different ages and allowed to live their whole lifespan were reanalyzed. We analyzed the shifting ages regarding the effect of age at exposure on cumulative mortality using data from the 1.9-Gy irradiation group [6], and the dose–response relationship using data from the day-0 [7] and day-7 irradiation groups [8]. In addition, we analyzed the shifting ages for ovarian tumor using tumor prevalence data [9]. Age-specific and site-specific cancer risks have not been reported except for ovarian tumors; thus, we selected ovarian tumors as a typical example of site-specific cancers. The details of the data sets used for this study are provided in Table 1.

**Table 1:** Details of full lifespan experiments in acute X-ray irradiated mice

References	Age at irradiation	Dose and dose rate	Number of mice (per group)	Age-specific cumulative mortality
[9]	Day 17 in utero, 0, 7, 35, 105, 240, 365, 550	0.1, 0.48, 0.95, 1.43, 1.90, 2.38, 2.85, 3.80, 5.70 Gy, 0.80–0.98 Gy/min	65–1888	^*^1
[8]	Day 7	0.1, 0.48, 0.95 Gy, 0.80–0.98 Gy/min	205–1003	^*^2
[6]	Day 17 in utero, 0, 7, 35, 105, 240, 365, 550	1.9 Gy	81–885	^*^3
[7]	Day 0	0.48, 0.95, 1.43, 1.90, 2.38, 2.85 Gy, 0.87 Gy/min	206–885	^*^2
[10]	Day 0	0.48, 0.95, 1.43, 1.90, 2.38, 2.85, 3.80, 5.70 Gy, 0.87 Gy/min	169–1878	Not assessed
[11]	Day 17 in utero, 0, 7, 35, 105, 240, 365	0.95, 1.9, 2.85, 3.8 and 5.7 Gy, 0.98 Gy/min	65–332	Not assessed
[12]	Day −2, 0, 7, 35, 105, 240, 365	0.95–5.7 Gy	–	Not assessed

The mice were B6C3F_1_ females in all experiments. Day 0 irradiation was performed within 24 h of birth. ^*^1: Only the prevalence of ovarian tumors; ^*^2: all causes of death; ^*^3: all causes of death and all solid tumors.

Age-specific cumulative mortality data for ovarian tumors have not been reported, although the age-specific prevalence of ovarian cancer has [9]. The age-specific prevalence in interval i of age (ρ_i_) was defined as the ratio of the number of mice that died with an ovarian tumor (N^*^_i_) to the total number of mice that died in interval *i* (N_i_): ρ_i_ = N^*^_i_/N_i_ [9]. Since ovarian tumor-related data include lethal and nonlethal cases, the number of deaths in each age period is required to estimate the cumulative prevalence of ovarian cancer. First, the number of deaths in each age period was calculated from the cumulative mortality [6–8], and the number of dead mice with ovarian tumor was calculated. Then, the cumulative number of dead mice with ovarian tumor was calculated for each age period. Subsequently, the cumulative prevalence for each period was calculated by dividing the cumulative number of dead mice with ovarian tumor by the total number of mice in the group. The results of cumulative prevalence are shown in Supplementary Fig. 1.

### Fitting the data of cumulative mortality or tumor prevalence to each attained age

**Fig. 1:**
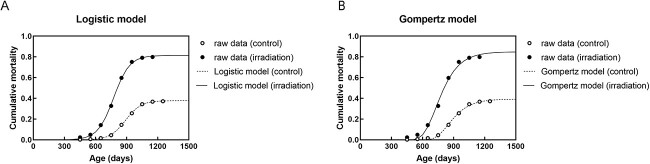
The models in this study. (A) Logistic model and (B) Gompertz model for cumulative mortality for all solid tumors in nonirradiated (control) mice and mice irradiated at 24 h of birth with 1.9 Gy.

First, the cumulative mortalities of mice with all solid tumors were fitted to a logistic model and the Gompertz model as a typical example of sigmoid curves, both of which are commonly used for assessing survival. Since a better fit was obtained using the logistic model in all data of cumulative mortality of all solid tumors, according to the Akaike Information Criterion using R software (Fig. 1 and Table 2), this model was adopted for subsequent analyses. The three parameters of the logistic model were calculated using the following formula:


$$ y=\frac{asym}{1+\exp \left(\frac{xmid-x}{scal}\right)}$$

where, *asym*, *xmid* and *scal* are coefficients of function of the logistic model and were obtained by SSlogis function in R software [14]. *Asym* is a numeric parameter representing the asymptote, and *ximd* is a numeric parameter representing the *x* value at the inflection point of the curve; thus, the value of the SSlogis function will be *asym*/2 at *xmid*. *Scal* is a numeric scale parameter on the input axis [14]. When the *asym* for cumulative mortality of all cause of death was greater than one, the *asym* was fixed at 1 and the other two parameters were calculated. In the case of cumulative mortality of all solid tumors and cumulative prevalence of ovarian tumors, the *asym* was fixed at maximum raw data and the other two parameters were calculated when *asym* was greater than the maximum raw data. The comparison of free *asym* and fixed *asym* is shown in Supplementary Figs 2–4, and the parameters are shown in Supplementary Fig. 5.

**Table 2:** Akaike information criterion values of the logistic model and Gompertz model for assessing cumulative mortality related to all solid tumors

Treatment	Logistic model	Gompertz model
Control	−66.3	−53.1
1.9 Gy-irradiation at Day 0	−43.4	−27.8
1.9 Gy-irradiation at Day 7	−40.6	−30.6
1.9 Gy-irradiation at Day 35	−43.5	−31.9
1.9 Gy-irradiation at Day 105	−42.1	−39.7
1.9 Gy-irradiation at Day 365	−66.3	−53.1

### Estimating shifted days

We analyzed the shift required in the number of days in age to match the irradiated curve and shifting the nonirradiated curve. The number of shifted days was determined when the residual sum of squares (RSS) between *y_estimated_* and *y_irradiated_* was at the minimum for each age point (*x*) of 250–1250 days depending on the data points of the data set [6–9].


$$ {y}_{estimated}=\frac{asym_{contol}}{1+\exp \left(\frac{xmid_{control}-\left(x- shifted\ days\right)}{scal_{contol}}\right)} $$

$$ {y}_{irradiated}=\frac{asym_{irradiated}}{1+\exp \left(\frac{xmid_{irradiated}-x}{scal_{irradiated}}\right)}$$

For all solid cancers and ovarian tumors, the shifting age of death was calculated after aligning the lifetime mortalities as follows:


$$ adjustment\ factor=\frac{asym_{irradiated}}{asym_{contol}} $$

$$ {y}_{estimated}= adjustment\ factor\times \frac{asym_{contol}}{1+\exp \left(\frac{xmid_{control}-\left(x- shifted\ days\right)}{scal_{contol}}\right)} $$

The suitability of the model was judged by the above-mentioned RSS.

## RESULTS

### Cumulative mortality by all causes of death

**Fig. 2:**
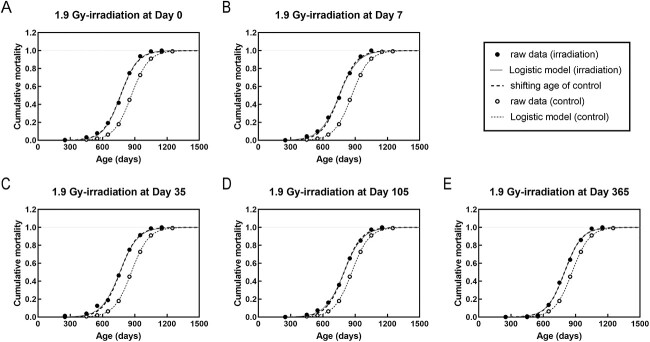
Cumulative mortality fitting curves for mice exposed to 1.9 Gy irradiation for all causes of death in the control (nonirradiated) and 1.9 Gy-irradiated groups. (A–E) Mice were irradiated at days 0 (A), 7 (B), 35 (C), 105 (D) and 365 (E). Circles are the raw data. Solid and dashed lines are data fitted by the logistic model. Dotted lines are the shifted lines to estimate the mortality of the irradiated group from the nonirradiated group.

**Fig. 3:**
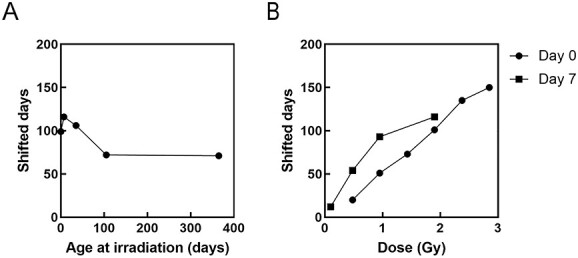
Dependence of shifted days on age at irradiation and dose for all causes of death. (A) Effect of age at exposure on the shifted days in response to 1.9 Gy irradiation. (B) Dose responses for irradiation at 0 and 7 days old.

Analysis of the cumulative mortality for all causes of death showed that the time of death in the irradiated group uniformly shifted, and thus was the same as the slope of cumulative mortality in the nonirradiated group (Fig. 2). Compared with the nonirradiated group, age at death shifted uniformly by 99–116 days in the groups irradiated with 1.9 Gy at 0, 7 and 35 days, and by 71–72 days in the groups irradiated with 1.9 Gy at 105 and 365 days of age (Fig. 3A and Table 3).

**Table 3:** Shifted days for all causes of death

Treatment	Shifted days
1.9 Gy-irradiation at Day 0	99
1.9 Gy-irradiation at Day 7	116
1.9 Gy-irradiation at Day 35	106
1.9 Gy-irradiation at Day 105	72
1.9 Gy-irradiation at Day 365	71

With respect to the dose response, for day 0 of irradiation, the number of shifted days increased in a dose-dependent manner (circles in Fig. 3B; *y* = 56.1*x* − 5.2, R^2^ = 0.993). Although a less linear dose response was observed for day 7 of irradiation (*y* = 55.0*x* + 21.7, R^2^ = 0.884), it appeared as a linear response when focusing only on mice irradiated with a dose of ≤1 Gy (*y* = 94.3*x* + 5.0, R^2^ = 0.994) (squares in Fig. 3B). Day-7 irradiation generally imposed a longer shifted days than day 0 irradiation. The shifted days per Gy varied depending on the age at irradiation such as 50 days by Day-7 irradiation and 94 days by Day-0 irradiation (Fig. 3B) for an average lifespan of approximately 860 days.

**Fig. 4:**
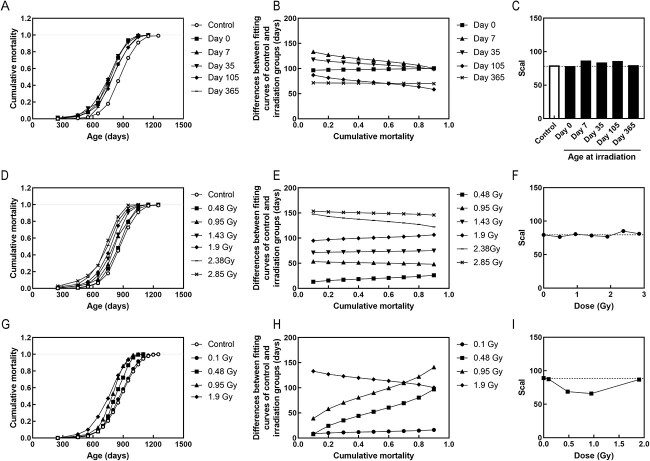
Detailed analysis of shifted days for all cause of death. (A–C) Effect of age at exposure on the shifted days in response to 1.9 Gy irradiation. (D–F) Dose responses for irradiation at 0 days old. (G–I) Dose responses for irradiation at 7 days old. (A, D, G) Cumulative mortality for all causes of death. (B, E, H) Differences between fitting curves of control and irradiation groups. The shift is considered uniform if the line is parallel to the x-axis, otherwise, it is considered non-uniform. (C, F, I) *Scal* indicate the slope of the cumulative mortality.

A more detailed analysis revealed that in some instances there was no uniform translation; thus, the differences in some data points of cumulative mortality between the fitting curves of control and irradiated groups were compared (Fig. 4). The irradiation at days 0 and 365 resulted in a uniform shift, which was not the case for irradiation at days 7, 35 and 105 (Fig. 4A and B). The number of shifted days decreased with increasing age at irradiation for 1.9-Gy irradiation at days 7, 35 and 105 (Fig. 4B). Moreover, a dose response analysis showed a generally uniform shift for all doses at day 0 of irradiation (Fig. 4D and E) and it again noted that the shifted days increased linearly as the dose increased (circles in Fig. 3B). At day 7 of irradiation, shifted days of the 0.1 Gy-irradiated group was little changed as a function of cumulative mortality against the control, but those of the 0.48- and 0.95-Gy irradiation groups changed significantly as cumulative mortality increased, and thus not uniformly (Fig. 4H and I).

### Cumulative mortality due to all solid cancers

**Fig. 5:**
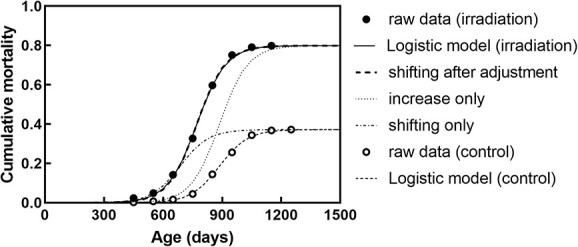
Explanation of curves of solid tumors. Representative examples of increasing only, shifting only and shifting after adjustment for Control group and 1.9 Gy-irradiated group at day 0.

As described above, the cumulative mortality for all causes of death in the 1.9 Gy-irradiated group could be determined by shifting the curve of cumulative mortality in the nonirradiated group with a uniform shift in the age (x-axis) (Fig. 2 and 4, Table 3), but shift was not always uniform, depending on dose and age at irradiation. In comparison with all cause of deaths, the solid tumors did not show a uniform shift (Fig. 5), possibly due to the different magnitude of the cumulative mortality (y-axis, lifetime mortality) within tumors. For example, for the 1.9 Gy-irradiated group at day 0, there was no consistency between irradiated-raw data (black circles in Fig. 5) and uniform shifting by 200 days (line of shifting only in Fig. 5). This is because the lifetime mortality doubled with irradiation at days 0, 7 and 35, and slightly increased by 1.2 folds after irradiation at days 105 and 365 (center row of Table 4). When we simply adjusted the lifetime mortalities by aligning the lifetime mortality of the control to that of irradiated group (line of increase only in Fig. 5), there was no consistency with the irradiated-raw data (black circles in Fig. 5). However, when we estimated the shifted days after adjusting the lifetime mortalities (line of shifting after adjustment in Fig. 5), there was good consistency. Consequently, we estimated the shifted days after this adjustment (Table 4). Compared with the nonirradiated group, the age of death shifted uniformly by 115–135 days in the groups irradiated with 1.9 Gy at days 0, 7 and 35, and shifted by about 56–80 days in the groups irradiated with 1.9 Gy at days 105 and 365 (Supplementary Fig. 6 and Table 4). Therefore, application of the concept of ‘shifting age of death’ after this adjustment showed a better agreement between the raw data of the irradiated group and the estimated curve. The response switching between the days 35 and 105 was similar to the mortality of all causes of death.

**Table 4:** Adjustment factor and shifted days for all solid tumors

Treatment	Adjustment factor (magnitude)	Shifted days
1.9 Gy-irradiation at Day 0	2.1	115
1.9 Gy-irradiation at Day 7	2.1	131
1.9 Gy-irradiation at Day 35	1.9	135
1.9 Gy-irradiation at Day 105	1.2	80
1.9 Gy-irradiation at Day 365	1.2	56

### Cumulative prevalence of ovarian tumors

**Fig. 6:**
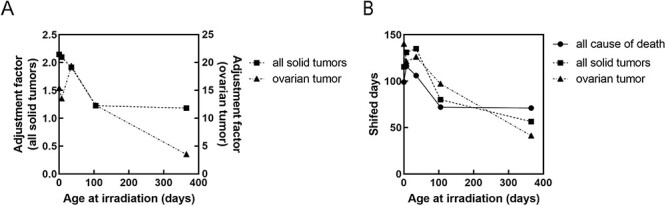
Summary of response of age at irradiation for assessing cumulative risk due to all causes of death, all solid tumors and ovarian tumors at 1.9 Gy irradiation. (A) adjustment factor. (B) shifted days.

Finally, to examine the shifting day of site-specific tumors, we analyzed data from mice with ovarian tumors, including lethal and nonlethal tumors, which were available for each age group (Supplementary Fig. 7). The cumulative prevalence of ovarian tumors required a greater adjustment factor compared with the analysis of all solid tumors (Fig. 6A). A response switch was also observed between days 35 and 105, similar to the cases of all solid tumors and all causes of death, although the degree of shifted days was smaller in the day 365 irradiation group (Table 5 and Fig. 6B).

**Table 5:** Adjustment factor and shifted days for ovarian tumors

Treatment	Adjustment factor (magnitude)	Shifted days
1.9 Gy-irradiation at Day 0	15.4	140
1.9 Gy-irradiation at Day 7	13.5	121
1.9 Gy-irradiation at Day 35	19.4	126
1.9 Gy-irradiation at Day 105	12.3	97
1.9 Gy-irradiation at Day 365	3.5	41

**Fig. 7:**
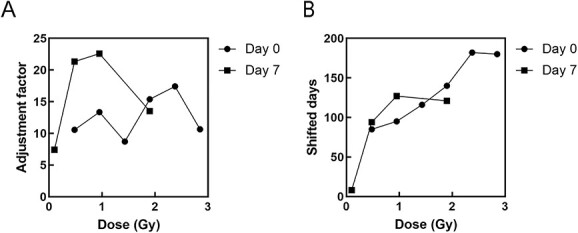
Dose responses for assessing cumulative risk due to ovarian tumors. (A) adjustment factor. (B) shifted days.

Considering the cumulative prevalence of ovarian tumors, the adjustment factors did not show a linear response (Fig. 7A), and shifted days reached saturation at high doses of 0.95 Gy for day 7 and 2.38 Gy for day 0 (Fig. 7B).

## DISCUSSION

For all causes of death, the cumulative mortality curves of irradiated groups were well-matched by uniformly shifting the curve of the nonirradiated group compared to all solid tumors and ovarian tumors. Although the curves for the cumulative mortality of all solid tumors and the cumulative prevalence of ovarian tumors did not match by simply shifting them uniformly, they became consistent after adjusting lifetime mortalities or prevalence. This may be due to the increased risk, which cannot be expressed in the model of earlier radiation risk, or changes of death causes (competition of death causes). This indicates that both discussions about the viewpoint of increase/earlier radiation risk and also the effects of competition for causes of death should be further explored.

As shown in Fig. 4, there were differences in the uniformity of the shift and the amount of days shifted in the irradiation groups according to irradiation conditions such as age and dose. For example, it is possible that they were not constant because the proportion of mice with pituitary and liver tumors increased in the group exposed to 1.9 Gy irradiation on day 0, and the proportion of mice with other solid tumors increased in the group exposed to 1.9 Gy irradiation on day 35 compared with the nonirradiated group [6]. Therefore, it is necessary to carefully discuss whether the concept of uniformly shifting age of death [2] can be applied to all irradiation conditions for the purpose of radiation protection and evaluating the cause of death and mechanism.

The differences in shifted days in all causes of death were milder compared with those in all solid tumors and ovarian tumors (Fig. 6B). This could be explained by the increase in cancer-related mortality, which reduces the number of deaths attributed to causes other than tumors and diseases due to competition for causes of death, and thus the overall effect may seem smaller when considering all causes of death. In addition, the shifted days for the 1.9-Gy irradiation group were longer in all solid tumors and ovarian tumors than in all causes of death. Therefore, we cannot add the risk of site-specific tumors for overall risk estimation.

For all causes of death, the irradiation at day 0 showed a linear dose response, but the irradiation at day 7 showed a linear dose response when limited to 1 Gy or less. For ovarian tumors, the number of shifted days reached saturation at high doses of 0.95 Gy and 2.38Gy for mice irradiated at day 0 and 7, respectively. Although the linear relationship may be applicable to shifted days in mice, it is necessary to further investigate whether the concept of ‘shifting age of death’ is applicable to radiation protection.

In the 1.9 Gy irradiation condition, the response was switched between 35 and 105 days consistently for all-cause mortality, all solid cancer mortality and ovarian cancer prevalence. Mouse ages of 0, 105 and 365 days correspond to human ages of 0, 20 and 30 years, respectively [15]. The responses switched at a maximum of 105 days of age (equivalent to about 20 years in humans), which supports different risks of irradiation exposure for adults and children [16]. Indeed, the young age period is considered to be a highly radiosensitive period, and thus, our results are consistent with this previous finding. Regarding the consideration of age in radiation protection, there is a possibility that a certain range of exposed ages can be considered, without focusing on a specific age and also without considering the parameter of attained age when applying the concept of ‘shifting age of death.’

One limitation of this study is that since only published data were used, the irradiation conditions that could be explored were limited. If further raw data become available in the future, it will be possible to expand the analysis range, such as the dose response of all solid tumors and dose rate effects. It is important to continue to collaborate among epidemiological, biological and computational studies on radiation risk evaluation of individual cancers. For example, computational studies can suggest a model to address the relationship between the competition for cause of death and adding site-specific risks to estimate whole risk in animal experiments. This can contribute to the analysis of irradiation conditions without modifiable factors, such as smoking and epidemiological studies, which have a huge advantage to estimate risk in humans.

## DATA AVAILABILITY

Data supporting the findings of this study are available within the article. The details of the data sets used for this study are provided in Table 1.

## Supplementary Material

shifting_age_suppl_info_rrad006
