# Supplementary material for: Effects of irradiation on cumulative mortality in mice: shifting toward a younger age of death
Source: J Radiat Res. 2023 Feb 10;64(2):412–9. doi: 10.1093/jrr/rrad006 (PMC10036085; doi:10.1093/jrr/rrad006)
Supplement: shifting_age_suppl_info_rrad006 [file shifting_age_suppl_info_rrad006.docx]

**Supplementary Information**

Effects of irradiation on cumulative mortality in mice: shifting toward younger age of death





Supplementary Figure 1. Cumulative prevalence of ovarian tumor

Age-specific cumulative prevalence was estimated using the data-set of ovarian age-specific prevalence [1] and corresponding age-specific cumulative mortality rate [2-4].





Supplementary Figure 2. Fitting of logistic model for all cause of deaths

The differences in fitting between free asym and fixed asym of 1 using the raw data-set of mice irradiated at Day 0 [2], that of mice irradiated at Day 7 [3], and that of mice irradiated at various ages [4].





Supplementary Figure 3. Fitting of logistic model for all solid tumors

The differences in fitting between free asym and fixed asym of 1 using the raw data-set of mice irradiated at various ages [4].





Supplementary Figure 4. Fitting of logistic model for ovarian tumor

The differences in fitting between free asym and fixed asym of 1 using the raw data-set [1].





Supplementary Figure 5. The results of parameters fitting by the logistic model

The three parameters of the logistic model were calculated using the following formula in R software:

$$y={asym}/\left[ 1+\text{exp}\left( \frac{xmid-x}{scal} \right) \right]$$

The break lines are the levels of control.





Supplementary Figure 6. Cumulative mortality fitting curves for all solid tumors

Cumulative mortality for solid tumors in the control group and 1.9 Gy-irradiated group on days 0, 7, 35, 105, and 365. The circles are raw data, lines of the logistic model are data fitted by the logistic model of fixed asym, and lines of shifting after adjustment are the shifted line to estimate the irradiated group by the non-irradiated group.





Supplementary Figure 7. Cumulative prevalence fitting curves for ovarian tumors

Cumulative prevalence for ovarian tumors in the control group and irradiated group. The circles are raw data, lines of the logistic model are data fitted by the logistic model of fixed asym, and lines of shifting after adjustment are the shifted line to estimate the irradiated group by the non-irradiated group.

**REFERENCES**

1. Sasaki S, Fukuda N. Dose-response relationship for induction of ovarian tumors in mice irradiated during prenatal, early postnatal and elder periods *J Radiat Res* 2008; 49: 623–33
2. Sasaki S, Fukuda N. Dose-response relationship for lifetime excess mortality and temporal pattern of manifestation in mice irradiated neonatally with gamma rays. *J Radiat Res* 2002; 43: 313–23.
3. Sasaki S, Fukuda N. Dose-response relationship for life-shortening and carcinogenesis in mice irradiated at day 7 postnatal age with dose range below 1 Gy of gamma rays. *J Radiat Res* 2006; 47: 135–45.
4. Sasaki S, Fukuda N. Temporal variation of excess mortality rate from solid tumors in mice irradiated at various ages with gamma rays. *J Radiat Res* 2005; 46: 1–19.
